# Mathematical Model of Dynamic Protein Interactions Regulating p53 Protein Stability for Tumor Suppression

**DOI:** 10.1155/2013/358980

**Published:** 2013-12-28

**Authors:** Hua Wang, Guang Peng

**Affiliations:** ^1^Mathematical Sciences, Georgia Southern University, Statesboro, GA 30458, USA; ^2^Department of Clinical Cancer Prevention, The University of Texas MD Anderson Cancer Center, Houston, TX 77030, USA

## Abstract

In the field of cancer biology, numerous genes or proteins form extremely complex regulatory network, which determines cancer cell fate and cancer cell survival. p53 is a major tumor suppressor that is lost in more than 50% of human cancers. It has been well known that a variety of proteins regulate its protein stability, which is essential for its tumor suppressive function. It remains elusive how we could understand and target p53 stabilization process through network analysis. In this paper we discuss the use of random walk and stationary distribution to measure the compound effect of a network of genes or proteins. This method is applied to the network of nine proteins that influence the protein stability of p53 via regulating the interaction between p53 and its regulator MDM2. Our study identifies that some proteins such as HDAC1 in the network of p53 regulators may have more profound effects on p53 stability, agreeing with the established findings on HDAC1. This work shows the importance of using mathematical analysis to dissect the complexity of biology networks in cancer.

## 1. Introduction

The tumor suppressor p53 is the master transcriptional regulator whose expression prevents the development of cancer [1]. Functional p53 expression is lost in about 50% of human cancer cases [2]. The MDM2 gene, a cellular protooncogene that is amplified in more than 7% of all human cancer cases [3], interacts with p53 and counteracts the tumor-suppressive function of p53 protein through various mechanisms, including blocking its transcriptional activity, exporting it into the cytoplasm and most importantly promoting its degradation [4].

MDM2 activities include those of a ubiquitin ligase, making it capable of targeting uniquitination of p53, which leads to p53 degradation [5, 6]. The ability of MDM2 to associate with and target p53 degradation depends highly on proteins that interact with MDM2 and p53, which provide an important mechanism of regulating p53 protein stability and expression [7].

The number of proteins implicated in regulation of p53 protein stability and degradation by modulating p53-MDM2 interaction is growing [8, 9]. By regulating this interaction, these proteins function as p53 degradation-promoting or -protecting molecules [10]. According to a categorized search of the literature using the IPA software program (Ingenuity Systems), 366 studies reported molecular regulators of p53 degradation, and 284 studies reported molecular regulators of p53 stabilization. These proteins participate in a variety of cellular processes, including transcriptional regulation, stress-response signaling, cell-cycle regulation, and metabolic process. Therefore, these proteins provide cells with diverse regulatory mechanisms for control of p53 protein expression in response to different cellular statuses. By positively or negatively regulating p53 expression, these proteins may suppress or promote tumor development, respectively.

*Genetic and Chemical Regulators of p53 Protein Stability and Degradation.* (i) Regulation of p53 stabilization (*n* = 284): MDM2, CDKN2A, TP53, NQO1, doxorubicin, EP300, MDM4, actinomycin D, deferoxamine, *γ*-radiation, DNA, TADA3, ultraviolet (UV) radiation, ionizing radiation, OTUB1, camptothecin, ATM, CHEK2, NQO2, 2-aminopurine, BAG6, BARD1, CP-31398, E1a, NUMB, SIN3A, disruption, myxothiazol, phosphotyrosine-binding domain, roscovitine, zidovudine, HSPD1, RB1, RPL23, USP7, oxidative stress, BANP, BRCA1, CAV1, CDKN2AIP, CDT, CP-257042, H-7, HIF1A, Hsp90, ING1, KRAS, MIR199A, PML, RPL11, RPL5, S100A4, TAF9, TP53BP2, TPP2, UVB radiation, WT1, X-ray radiation, ZNF668, benzyloxycarbonyl-Leu-Leu-Leu aldehyde, bisindolylmaleimide, bortezomib, cardiomyocytes, fludarabine, hydrogen peroxide, hypoxia, pX, radiation, reactive oxygen species, romidepsin, stress, 2-methoxyestradiol, 26s proteasome, 5-fluorouracil, ATF3, ATP, ATR, BAX, CCND1, CDB3, CDK2, CDK5, CRE-like element, CSNK2A1, DHRS2, DMTF1, E2F1, E6, GADD45A, GNL3, HSPA1A/HSPA1B, HTT, HUWE1, Hct 116 cells, Hsp27, IRF1, KDM6B, MAPK14, MYB, MYC, Mdm2 protein-binding domain, NAA30, ONCOGENE, PD98059, PHA-533533, PIN1, PLK1, PLRG1, POLR1A, PRKDC, PTEN, RB1CC1, RFWD2, RNA polymerase II, *S*-nitrosoglutathione, TERT, TGFB1, TOPORS, UIMC1, UVC radiation, VRK1, YWHAG, YY1, anoxia, caffeine, carboxy terminal domain, cisplatin, clasto-lactacystin *β*-lactone, cobalt chloride, cyclophosphamide, dicumarol, etoposide, genotoxic stress, methylnitronitrosoguanidine, mir-122, mitomycin C, mitoxantrone, morphine, nutlin-3, sulforafan, trichostatin A, ursodeoxycholic acid, and vincristine.

(ii) Regulation of p53 degradation (*n* = 366): MDM2, TP53, E6, ubiquitin, dicumarol, benzyloxycarbonyl-Leu-Leu-Leu aldehyde, COPS5, NQO1, UBE3A, MDM4, 26s proteasome, CDKN2A, CAT, E1b, zinc finger, C3HC4 type (RING finger), 20s proteasome, DHRS2, EIF2AK2, RAD23A, curcumin, PIM1, RAD23B, TOPORS, WR 1065, digoxin, etoposide, leptomycin B, ouabain, protein, zinc finger domain, ABL1, ATF3, Ala-Ala-Phe-chloromethylketone, CTNNB1, EIF2AK3, GSK3B, HTT, HUWE1, Jnk, NOTCH1, PSMD10, RB1CC1, RBBP6, RFWD2, TSG101, TXN, Ube3, YY1, dexamethasone, dsRNA, geldanamycin, lactacystin, monorden, stress, 6,4′-dihydroxyflavone, AKT1, ARRB2, ATP, AURKA, Akt, BANP, BCAS2, CAPN1, CSN, CUL2, CUL4A, CUL5, CUL7, E1a, E4orf6, EGTA, EP300, FBXW8, HDAC1, HIF1A, human adenovirus type 12, human adenovirus type 5, IKBKB, IKBKG, KAT5, LA-12, LDL, LY294002, large T antigen, Lmp1, MAGEC2/MAGEC3, MAPK1, MAPK3, Mageb, *N*-carbobenzyloxy-leucine-leucine-norvalinal, NAMPT, NCL, NF2, NQO2, P38 MAPK, PIM2, PKA, PML, POLR1A, PSMC1, PYR-41, RBX1, RFFL, RNF34, RPL11, SMAD1, SMURF1, STUB1, SYVN1, TCEB1, TPT1, TRIM28, UBE2D1, UBE2E1, UBIQUITIN LIGASE, UBQLN1, UBQLN2, Ubch5, ubiquilin, XPO1, Z-Leu-Leu-Leu-B(OH)2, ZNF668, *β*-estradiol, bortezomib, calpeptin, chrysin, cycloheximide, epoxomicin, esculetin, hypericin, nitric oxide, okadaic acid, roscovitine, and warfarin.

As reported in the literature, investigators have conventionally studied the regulatory effects of these hundreds of proteins as isolated molecular events, assessing their effects on p53-MDM2 interaction individually. However, the human biosystem is highly dynamic and complex. How the interconnected regulatory network formed by these p53-regulating proteins affects the functions of individual proteins is an intriguing question. The answer to this question is not only important for us to understand how dysfunctional p53-regulating network contributes to tumor initiation and progression, but also important for us to identify potential mechanisms to rewire this dysfunctional network in cancer.

To answer this question, we performed a network analysis using the IPA software program to identify any potential molecular networks that may be formed by known p53 protein regulators.

We selected nine candidate proteins among the hundreds of p53 regulators as representative examples of p53 protein stability-regulating network (Table 1). Their interactions with p53 and MDM2 have been well established in a series of studies [11–19]. We chose these proteins to form the system for our simplified mathematical model because they have well-established functions in cancer biology and a broad impact on regulation of both genetic and epigenetic processes. For instance, BRCA1 deficiency predisposes women to the development of breast and ovarian cancer [20]. Also, Rb1 is the key cell-cycle regulator, and deficiency of it promotes tumorigenesis in several human cancers [21]. Furthermore, like transcriptional regulators such as SMAD1, YY1, and WT1, HDAC1 functions as an epigenetic regulator of histone modification induced by acetylation [14–16]. In addition to their biological backgrounds, these nine proteins also correspond to critical nodes in the network structure generated with all proteins. Some of these nodes are cut-vertices (nodes in a graph/network whose removal disconnects the graph) and others are crucial to the connectedness (graphs/networks loses strong connectivity after removing these nodes).

**Figure 1:**
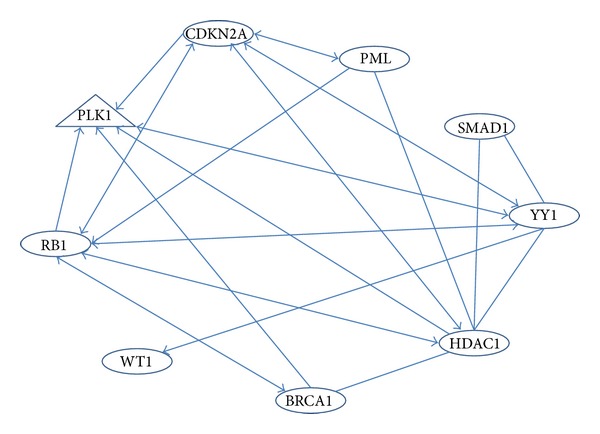
The network formed by p53-regulatory proteins.

As we expected, these proteins involved in regulation of p53 protein expression form a complex network with interconnected regulatory linkages (Figure 1). This simplified version of the network consists of only 9 representative candidate proteins; the entire network of hundreds of p53 regulators is expected to be much more complex.

As described above, numerous proteins interact with p53 and/or MDM2. In order to develop a systematic study of interactions among proteins in a massive network, determination of how several proteins in a network interact with p53 is the first logical step. For example, protein *A* may interact with p53, and protein *B* may interact with MDM2. If both *A* and *B* are present, however, their interactions with p53-MDM2 are far more complicated than a simple linear sum of or difference in their individual interactions. For example, the presence of *A* may enhance or inhibit the presence of *B*.

Although the importance of this study is evident, there is a surprising lack of consideration of this question. One simple way to describe a large graph (with many “nodes” and “edges”) is to assume that it can be approximated by a random-like structure [22]. As described herein, we tested a random walk model that presents a network of proteins as a directed graph with a restarting point and used stationary distribution to predict the potential impact of each protein in this network on p53-MDM2 interaction.

## 2. Background and Methodology

In this model, a directed graph is constructed with each protein represented by a node and each protein-protein interaction represented by a directed edge between the nodes corresponding to the proteins. An additional initial node (*S*) and an additional transition node (*T*) are added to the graph, such that *S* has directed edges to and from all existing nodes and *T* has directed edges from all existing nodes and a directed edge to *S*. Note that *S* and *T* are the artificial nodes that are independent of the rest of the network nor p53 and MDM2: *S*, with directed edges to and from all nodes, represents the impact on the network from other proteins and the receiver of the impact from the network (through *T*); *T*, with directed edges from all nodes and to *S*, represents the pathway through which the impact from the network is converted to other proteins. With this setup, the model can be applied to any networks that we wish to consider.

A random walker starts from the initial node. At each step, the walker moves along the directed edges to a neighboring node with equal probabilities. That is, if a node *A* has directed edges to *B*, *C*, and *D*, the random walker, when at *A*, will move to each of *B*, *C*, and *D* with probability 1/3. At each node, the directed edge from it to the transition node serves as the chance of exiting the random walk to external proteins. Also, the directed edge from the initial node serves as the chance of restarting this random walk, representing the impact from external proteins outside of this network. Clearly, the higher the probability of a node being visited by the random walker is, the more interference the corresponding protein contributes to the network.

With a total of *n* nodes in the directed graph, *p*_*i*_ denotes the probability of the random walker being at the *i*th node. The vector *P*_*t*_ = (*p*_1_, *p*_2_, …, *p*_*n*_) is then the “state” after *t* steps. The sequence of *P*_*t*_ as *t* goes to infinity (i.e., the random walker keeps walking forever) forms a Markov chain. The states are also called the transition probabilities.

Generally speaking, the necessary and sufficient conditions for convergence of Markov chains are “irreducible” [23] and “aperiodic” [24]. In simpler terms, equivalent condition are as follows.

- The graph is “strongly connected,” that is, every node has a directed path towards another.
- The greatest common divisor of all cycle lengths is 1.

We claim that both of these conditions are satisfied in our constructed network. First, the initial node *S* has a directed edge (hence, a directed path) to and from every other nodes in the graph. For any pair of nodes *A* and *B*, *A* → *S* → *B* → *S* → *A* provides the necessary directed paths from *A* to *B* and vice versa. Thus, the graph under consideration is strongly connected. Second, for any node *A*, the directed cycle *A* → *T* → *S* → *A* is of length 3. Also, for any directed edge *A* → *B*, the directed cycle *A* → *B* → *T* → *S* → *A* is of length 4. Therefore, the greatest common divisor of all cycle lengths is 1.

When a Markov chain converges, it has a unique limit. In our terms, the transition probability *P*_*t*_ converges to a unique vector *P* as *t* approaches infinity. This limit *P* is the unique “stationary probability” or “stationary distribution.” Such a convergence indicates that if the random walking process goes on forever, the probability of each node (protein) being visited (i.e., influencing the network) is a fixed value.

Using *M* to denote the adjacency matrix of the directed graph with edge weights corresponding to the probabilities (known as the transition matrix of this random walk), the stationary distribution can be directly determined by solving *PM* = *P*. In other words, an eigenvector of the matrix *M* − *I* transposed, in which *I* is the identity matrix.

As described above, we take *P* as the vector of the impact of this network on p53 and/or MDM2 expression. That is, let *P* = (*p*_1_, *p*_2_, …, *p*_*n*_), in which the first entry corresponds to the initial node and the last corresponds to the transition node. We take *X* to be the normalized vector of (*p*_2_, *p*_3_, …, *p*_*n*−1_); that is,

(1) $$\begin{array}{c} X=\frac{\left(p_{2},p_{3},\ldots ,p_{n-1}\right)}{N}, \end{array}$$

in which *N* is the norm ∑_*i*=2_^*n*−1^ *p*_*i*_ of (*p*_2_, *p*_3_, …, *p*_*n*−1_). The resulting vector *X* = (*x*_2_, *x*_3_, …, *x*_*n*−1_) provides a measure of how each node interacts with p53 and/or MDM2. Among these nodes in the network corresponding to *x*_2_, *x*_3_, …, *x*_*n*−1_, individual interactions of some of them with p53 and/or MDM2 are not necessarily present. Their corresponding *x* values therefore denote the percentages of these interactions contributed to the network.

**Figure 2:**
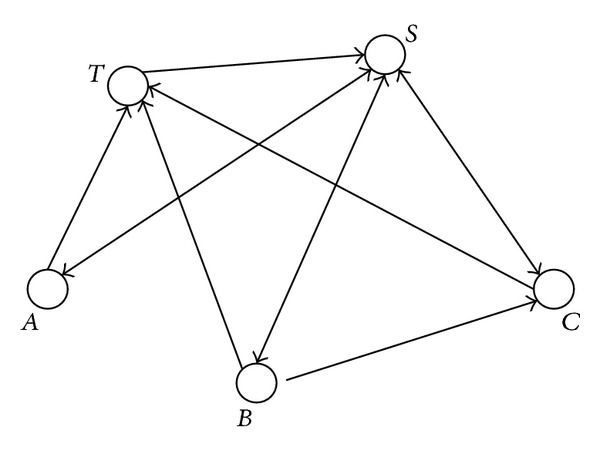


Figure 2 is a simple network having three original nodes—*A*, *B* and *C*—with one directed link from *B* to *C*, and the initial node *S* and transition node *T* together with the corresponding edges.

The corresponding transition matrix is

(2) $$\begin{array}{c} M=\left[\begin{array}{ccccc} 0 & \frac{1}{3} & \frac{1}{3} & \frac{1}{3} & 0\\ \frac{1}{2} & 0 & 0 & 0 & \frac{1}{2}\\ \frac{1}{3} & 0 & 0 & \frac{1}{3} & \frac{1}{3}\\ \frac{1}{2} & 0 & 0 & 0 & \frac{1}{2}\\ 1 & 0 & 0 & 0 & 0 \end{array}\right]. \end{array}$$

Solving *P* = *PM* yields the non-zero solution *P* = (1, 1/3, 1/3, 4/9, 1/2). Following the above calculations, we have *X* = (0.3, 0.3, 0.4), in which the first 0.3 corresponds to *A*, the second to *B*, and 0.4 corresponds to *C*. Thus, 30% of the individual interactions of each of *A* and *B* with p53 and/or MDM2 are effective in this network, whereas 40% of that of *C* is effective. Note that *A* and *B* had no direct or indirect interaction in the original network (without *S* and *T*). Thus, the same percentage for them makes perfect sense.

## 3. Results

The proposed method was applied to the network shown in Figure 1. With the addition of *S* and *T*, the resulted network consists of 11 nodes with a transition matrix of dimension 11 × 11:

(3) 00.14290.20000.25000.12500.11110.20000.50000.14290.33331.00000.111100.200000.12500.1111000.1429000.11110.14290000.1111000000.11110000.12500.1111000000.11110.142900.250000.1111000.14290.333300.11110.14290.20000.25000.125000.200000.1429000.111100000.1111000.1429000.11110000.12500000000.11110.14290.200000.12500.11110.200000000.11110.1429000.12500.11110.200000.14290000.14290.20000.25000.12500.11110.20000.50000.14290.33330.

Taking the eigenvector with respect to eigenvalue 1, we have

(4) (.7078,.2041,.1376,.1439,.2842,.2677, .1411,.1142,.2288,.2340,.3539)

as the stationary distribution. By taking away the first and last entry (which correspond to the artificial nodes *S* and *T*) and normalization, we have

(5) (.1163,.0784,.0820,.1619,.1525, .0804,.0650,.1303,.1333)

corresponding to the percentage of impact that each of the nine protein has on the entire network. The result yields a percentage of 15.25% for histone deacetylase 1 (HDAC1). Compared with the average value of 11.1% for any gene in the network, it suggests the importance of HDAC1 in its role of influencing p53-MDM2, not just as an individual gene, but also as part of a network with other genes. Indeed, HDAC1 has an enzymatic function to remove acetylation modification from proteins such as histone protein and p53 [25]. It also interacts with many epigenetic regulators such as chromatin remodeling factors, histone modification factors, and DNA methylation enzymes [26]. Thus it has a broad effect on cell growth, arrest, differentiation, and death via regulating epigenetic process [27]. These diverse biological functions of HDAC1 may explain its importance as a key p53 regulator implied through our mathematical analysis.

## 4. Discussion

The balance of p53 and MDM2 interaction with degradation-promoting and -protecting proteins eventually dictates p53 stability [8]. As we describe herein, this balance is likely affected by the complex array of protein interactions rather than a single molecular event mediated by an individual protein. Because of the complexity of the real biological system of cells, it is virtually impossible to experimentally dissecting and analyzing the common outcome of the protein network involved in regulation of p53 expression. Therefore, a mathematical model that can be used to analyze the common biological effects of the protein network on regulation of p53 protein expression is urgently needed.

In the present study, we used the random walk procedure with directed graphs to determine the comprehensive effects of the protein regulatory network on p53-MDM2 interaction. Simply put, the main advantage of this procedure lies in the fact that the stationary distribution can be instantly solved (hence, with minimum complexity) as an eigenvector of the transition matrix generated by the given network. The larger the corresponding value of a node in the stationary distribution is, the more likely the corresponding protein's individual interaction with p53 and MDM2 takes effect as part of the network.

p53 is an attractive therapeutic target for cancer because its tumor-suppressive activity can be stimulated to eradicate tumor cells [9]. Inhibiting physical p53-MDM2 interaction is a promising approach to reactivating p53. Therefore, this study may provide a general paradigm that outlines the interplay among known proteins interacting with p53-MDM2 and regulating p53 stability. Mathematically modeling the protein interactions that control p53-MDM2 interaction may provide biological insight into how the balance of their interacting proteins can be changed to ensure fine-tuned regulation of p53 stability.

In addition, to prevent tumorigenesis, molecular mechanisms similar to the p53 protein regulatory network can be found in the cellular systems that regulate other key tumor suppressors, such as PTEN. PTEN expression is lost in more than 50% of tumors [28]. However, PTEN mutations are not common in tumors [29]. Instead, dysfunctional regulation of PTEN expression at the protein level is a common initiating event in cancer development [29]. As mentioned earlier, the use of nodes *S* and *T* enables one to examine networks with any number of nodes. Therefore our mathematical model can be potentially used to examine additional molecular events in tumor suppression [30].

## 5. Conclusion and Future Work

In our study, we applied mathematical methods and concepts including random walk and stationary distribution to dissect the complexity of protein network in cancer biology. Our result shows that these methods can be used to identify key nodes in the protein network, which may not be readily determined by biological experiments studying individual protein as an isolated molecular event. This approach provides an advantage to analyze the functions of a group of genes or proteins as a network in cancer cells, which is not only important for our understanding of cancer etiology, but also for developing new therapeutic strategies.

p53 regulators identified by the IPA software program (Ingenuity Systems) from existing biology studies are involved in a variety of molecular pathways, that form diverse functional groups such as gene expression, DNA repair and replication, cell death and survival, cellular development, carbohydrate metabolism, cell-to-cell signaling and interaction, cell cycle, cell-mediated immune response, and posttranslational modifications. To fully realize the potential of the proposed model, we will (as a referee kindly suggested) apply the discussed method to networks of reasonable size formed by groups of proteins (according to the similarity of their functions). After analyzing each of these networks and choosing the proteins of highest scores (from each network resp.), the method can be applied again to the network formed by the chosen proteins. We expect very meaningful results through this approach when we take the time to complete the massive inputting and calculation.

## Figures and Tables

**Table 1:** Candidate p53-regulatory proteins.

Protein	
BRCA1	Breast cancer 1, early onset
HDAC1	Histone deacetylase 1
PLK1	Polo-like kinase 1
PML	Promyelocytic leukemia
CDKN2A	Cyclin-dependent kinase inhibitor 2A
SMAD1	SMAD family member 1
WT1	Wilms tumor 1
RB1	Retinoblastoma 1
YY1	YY1 transcription factor
